# Book Review: The Psychologist's Companion (6th Edition)

**DOI:** 10.3389/fpsyg.2019.00556

**Published:** 2019-03-21

**Authors:** Helena Schmiedl, Martin G. Köllner

**Affiliations:** Human Motivation and Affective Neuroscience Lab, Department of Psychology, Institute of Psychology, Friedrich-Alexander University Erlangen-Nürnberg (FAU), Erlangen, Germany

**Keywords:** academic psychology, academic success, research practices, PhD students, undergraduates, writing skills

The sixth edition of *The Psychologist's Companion* (Sternberg and Sternberg, 2016) is a guide to successful work in psychology, be it as a student, teacher, or researcher. It intends to accompany you all the way throughout developing, conducting, and reporting a psychological experiment. The book focuses on tips about the writing process, with a pronounced focus on papers in scientific psychology. The coverage ranges from basics like idea generation or literature research, to formal and data presentation guidelines, up to preparations for journal submissions. A separate section is concerned with successfully presenting oneself in academia, including (grant/book) proposals and lectures, or, in advanced career stages, applications for a professorship and communicating with the press. Looking at the overall selection of content, *The Psychologist's Companion*, like in its earlier editions, remains a valuable reference for students and scientists, now expressly also addressing teachers in its title.

Since the book addresses mainly psychology students and researchers, most parts are written as a step-by-step manual to guide the reader through different tasks that occur during an experiment. Due to the mostly chronological structure of topics that occur in the process of a study (designing informed consent forms and debriefing sheets, obtaining institutional review board approval, or the like), it can easily be used as additional reading material for practical experimental psychology courses. Correspondingly, we have employed it successfully as accompanying literature in our undergraduate experimental psychology and field research practica. Newly added to this edition are three categories of boxes to further the learning process and encourage readers to look deeper into some points the authors make, including stories from the authors' personal experience or questions requiring the reader to apply the just learned facts. With several easy-to-use and helpful checklists one can make sure not to have overlooked anything important. Because of this structure, *The Psychologist's Companion* not only offers the theoretical input but also the practical applications needed to keep students interested.

However, there are some limitations. Open Science (e.g., Yamada, 2018)—not even listed in the subject index—is not substantially featured as a coherent part of the book's content. In fact, aside from open access publishing, after careful reading we could find only a brief reference without explanation to core topics like preregistration and open materials/data on p. 259 as a possibility when submitting to *Psychological Science*. Considering the outcomes of the Replication Crisis (overview in Schultheiss and Mehta, 2018) and the resulting movement in psychological research, this is no longer appropriate for a guide addressing the needs of students and researchers. Even more, some passages, if taken literally by students during their first experiences with running experiments, may mistakenly be interpreted as encouraging Questionable Research Practices (QRPs, explained e.g., in Bakker et al., 2012). This is exemplified in statements like calling it a myth that researchers have their to-be-tested ideas ready before data collection and stating that “One's ideas [about the outcome] develop along with the experiment” (p. 23), despite the fact that preregistration has become a widespread practice in our discipline. In addition, it is not helpful to instruct students on the same page that data sets can be analyzed in an infinite number of ways and that the method that “yield[s the] maximum payoff” should be selected, when nowadays analyses are registered prior to conducting the study and *p*-hacking (Wicherts et al., 2016) is an acknowledged problem. This is not beneficial to the authors' actual intentions, as for example the book clearly recommends the planning of major data analyses in advance and repeatedly mentions the need to label *post-hoc* hypotheses as *post-hoc*. Thus, the authors should consider clarification and incorporating the practices detailed for example in Yamada (2018) in future editions. Especially the chapter on *Planning and Writing the Experimental Research Paper* should include at least an overview of the subject and the associated procedures. Also, some recurring tips like proofreading one's work before submitting it should be common sense and therefore don't need to be mentioned repeatedly. While, altogether, this is a useful and important book, it should be handled with care when used as in-class material. It is essential to give additional instructions to students regarding current research practices to avoid misunderstandings.

In comparison to other books on similar topics, this one has several advantages. While *Writing Your Journal Article in 12 Weeks* (Belcher, 2009) offers a detailed chronological to-do-list and even prepared sheets to fill in your progress, it is not specially written for psychologists. Furthermore, it expects the user to already have a general idea of the topic or even a prewritten script to work on, so it doesn't really apply to first-year university students. *The Psychologist's Companion* therefore is most helpful for beginners, especially for students writing their first paper or conducting their first experimental study. It also focuses on thematic steps whereas Belcher's book provides exact time templates.

In summary, the book can still be recommended especially to early-career researchers starting their PhD studies and to undergraduate students. The latter group may consider *The Psychologist's Companion for Undergraduates: A Guide to Success for College Students* (Sternberg and Sternberg, 2017), which however bears a striking resemblance to the present book in topics covered and is almost identical over the course of large passages. As a final note, in the light of the present book's missing coherent incorporation of modern research transparency, some limitations and potentially misleading passages need to be made explicit to the students and to be supplemented by additional instruction on current research practices.

## Author Contributions

HS and MK wrote the manuscript, with larger contributions by HS. MK then provided edits and suggestions for revision.

### Conflict of Interest Statement

The authors declare that the research was conducted in the absence of any commercial or financial relationships that could be construed as a potential conflict of interest.
